# An Account of Earned Forgiveness through Apology

**DOI:** 10.1007/s11406-017-9868-2

**Published:** 2017-07-15

**Authors:** Cristina Roadevin

**Affiliations:** Department of Philosophy, University of Sheffield, 45 Victoria Street, Sheffield, S3 7QB UK

**Keywords:** Forgiveness, Apology, Respect, Resentment

## Abstract

I start by presenting an intuitively appealing account of forgiveness, ‘the insult account’, which nicely explains the cycle from wrongdoing to forgiveness. We need to respond to wrongdoing by blaming our offenders because they insult us with their actions (Murphy 1988; Hieronymi *Philosophy and Phenomenological Research, LXII*(3), 529–55, 2001; Hampton 1988a, b). How can wrongdoing be overcome? Either by the retraction of the insult or by taking necessary steps to correct for the wrong done. Once the insult has been retracted, usually by apology or remorse, forgiveness can come about. Martin *The Journal of Philosophy, 107*(10), 534–53, (2010) has recently criticized this promising account of forgiveness. My aim here is to defend an improved version of the ‘insult account’. I propose an account of *earned forgiveness through apology*, which shares features with the ‘insult account’ criticized by Martin, but also improves upon problems found in the ‘insult account’. This new account will successfully solve the puzzle of forgiveness. Drawing on Bovens’ (2009) account of apologies, I argue that apologies uniquely earn the wrongdoer’s forgiveness. I finally address a concern about the relation between apologies and forgiveness, recently raised by Hallich *Ethical Theory and Moral Practice, 16*(5), 999–1017, (2016). I argue that my expressive view of what the function of apologies is will answer his skepticism about apologies.

## Introduction

In this paper I take the view that when we forgive, we make a commitment to put the offense behind us and move on, by ceasing to hold that particular wrong against the wrongdoer, and eventually overcoming hurt feelings that the offense has caused us.^1^ In forgiving, we further give the offender an opportunity not to repeat the moral offense. So forgiveness involves two kinds of expectations: the victim is expected to put the wrong behind—this may involve not bringing it up again in future conversation in a blaming manner, overcoming blame feelings and attitudes concerning that particular offense, or maybe replacing negative feelings with more positive feelings towards the wrongdoer, such as trust or good will. On the other hand, the wrongdoer is trusted not to do that same thing again and she is therefore held responsible for not repeating the bad behaviour.

The question I try to answer here is what exactly can both justify and earn the victim’s forgiveness. In answering this question, I draw a distinction between *being justified* and *being earned.* Many things can justify forgiveness (such as compassion and self-preservation, in cases of elective forgiveness^2^) but the claim I will argue for is that only apology can earn it. Thus, uniquely through apology, forgiveness can be justified by being earned.

It is worth clarifying right from the start that I’m interested here in an account of ‘redemptive forgiveness^3^—or what I call ‘earned forgiveness’—whereby forgiveness is made rational by the reparative activity of the wrongdoer. However, I’m aware that in doing this I neglect another important type of forgiveness, elective forgiveness, which is offered as a gift to unapologetic wrongdoers. I’m not committed here to the claim that one type of forgiveness is more valuable or common than another. I’m happy to admit that elective forgiveness is equally valuable in enabling the victim and the wrongdoer to move on with their lives.^4^ However, my concern in this paper is with an account *of earned forgiveness through apology*, and, in particular, with the question of why apologies provide the victim with a reason to forgive.

In explaining the role that apologies have in justifying forgiveness, many authors have argued that forgiveness involves a certain puzzle. That is, one must not only explain what justifies a victim to cease to hold a particular wrong against a wrongdoer and eventually overcome blame feelings toward her, but also how a victim can do all this without denying that her wrong was an unexcused and unjustified moral offence.

Pamela Hieronymi (2001) and Jeffrie Murphy (1988; 2003) argue that we can solve this puzzle of forgiveness if we consider the fact that our offenders, when they wrong us, insult and degrade us with their actions. According to Hieronymi and Murphy, wrongdoing communicates to others a certain insulting message: that it is acceptable to treat the victim in this undignified way and that such treatment is acceptable in general (Hieronymi 2001: 546). Wrongful deeds therefore communicate messages about what the victims deserve, but also about their moral status. Resentment, on their view, is a form of protest against this insulting message. Apologies work in relation to forgiveness by in effect retracting the insult. Apology justifies the relinquishing of our resentment so now forgiveness can be forthcoming. I will follow Adrienne Martin’s (2010) practice of referring to this as the *insult account.*

Martin (2010) has recently criticized this promising view of forgiveness and apology. Moreover, I agree with her that the particular strand of the ‘insult account’ she criticizes is unsatisfactory. However, it is important to preserve the insight behind this account, which is that what is essential to understanding apology and forgiveness is the fact that wrongdoing disrespects us as agents. I put forward a new version of the ‘insult account’, *an account of earned forgiveness through apology*, which can solve the puzzle of forgiveness and explain the unique role of apologies in earning forgiveness. The account I propose also challenges the widely defended claim that forgiveness involves the total overcoming of resentment. It will follow from my analysis of apology that a victim can forgive even if her ‘heart’ has not yet caught up with how she feels about the wrongdoer.^5^ I argue that the purpose of earned forgiveness is to acknowledge that the wrongdoer has made good the disrespect expressed by her wrongdoing through apology, followed by a commitment to put the wrong behind and eventually repair relations.

I finally address a concern about apologies in general, recently raised by Hallich (2016). I argue that my expressive view of what the function of apologies is will answer his skepticism about apologies.

## The Puzzle of Forgiveness

Let me start by discussing the puzzle of forgiveness, formulated by Hieronymi (2001) and Allais (2008). On the one hand, these accounts argue that forgiving implies that we put the wrong behind us—we commit to letting go of negative feelings toward the wrongdoer, such as resentment and anger.^6^ On the other hand, the space for forgiveness opens when we see our wrongdoer as culpable for what she has done—when we can legitimately blame our offender for hurting us. Forgiveness thus requires continued moral condemnation of the wrong. Nevertheless, if this is the case—if it is necessary to always acknowledge the culpability of the wrongdoer, even after one has forgiven—then it is difficult to see how the victim can put the offence behind her and forgive the wrongdoer without relinquishing justified blame feelings. Let me reformulate the puzzle. It seems that forgiveness only makes sense when we accept the following preconditions:

1. What the wrongdoer did was wrong; it was a moral offence. (If there is no moral offence, then there is nothing to forgive the wrongdoer for.)
2. The wrongdoer is in general capable of being responsible for her actions. (If the wrongdoer lacks capacities for responsibility, then, again, there is nothing to forgive because it would be unfair to blame such a person.)
3. The wrongdoer is responsible for this particular wrongdoing, so she is not to be excused for what she did—excusing is not the same as forgiving.^7^

The problem for a theory of forgiveness is to show how it is possible to forgive the wrongdoer, while continuing to hold her responsible for what she did. The challenge is therefore to explain how one can forgive without denying at least one of the three preconditions listed above, despite the fact that they are the very preconditions that make appropriate our blame feelings against the wrongdoer.

The puzzle can be solved, some writers have argued,^8^ if the wrongdoer takes steps to correct for what she has done by apologizing to the victim. It seems that even if an offender has wronged us seriously, we are often willing to forgive her if she sincerely apologizes. This might seem puzzling as it is unclear why such a simple gesture can have the power to convince the victim to grant forgiveness to her wrongdoer. It is therefore crucial for a theory of forgiveness to explain how apologies work in relation to forgiveness. Apologies have the power to provide the victim with the right moral reasons to forgive her wrongdoer. Admittedly, forgiveness may also come about without apology where forgiveness is offered as a gift to the wrongdoer, and, furthermore, sometimes apologies may be given yet forgiveness is not forthcoming.^9^ Despite these examples, there remains a justificatory connection between sincere apologies and earning forgiveness,^10^ and any account must explain why apologies have reason-giving powers.

I turn now to discuss an influential account in the literature of forgiveness, the ‘insult account’, which seems to solve the puzzle of forgiveness and explain the connection between apology and earned forgiveness.

## The ‘Insult Account’

According to the ‘insult account’ of forgiveness, when a wrongdoer wrongs us, she communicates to the victim that his moral worth does not preclude such treatment: ‘I count and you do not’ (Murphy 1988: 28), or ‘You can be treated in this way’ (Hieronymi 2001: 546). It follows that, according to this view, the mere fact that someone wrongs us will insult and undermine our worth.

Hampton (1988a, b) criticizes Murphy’s claim that wrongdoing insults us and sometimes successfully degrades us. Hampton argues that insults can be understood in two ways, as ‘demeaning’ or ‘diminishing’. She argues that we can demean someone by wronging them without actually diminishing them or, in Murphy’s wording, degrading them—the victim might not feel that their sense of worth has been degraded. Hampton argues that we can wrong victims by insulting them, even if the victims are not and do not feel diminished in any way. For Hampton, victims cannot strictly speaking be diminished, even if they feel diminished, because moral status is always equal and not something which can be lowered. I will return to this distinction because it will be important for the account I want to defend. I agree with Hampton that actions can insult wrongdoers, but without diminishing them.

Hieronymi, contrary to Hampton, understands insults to our sense of worth (in the ‘diminishing sense’) in terms of what she calls ‘the threatening claim’ which the wrongful ‘event’ makes. Following from this, Hieronymi proposes to understand resentment as a form of protest against the ‘threatening claim’ made by the wrongdoer’s action, a ‘claim’ which causes the victim to feel threatened and thereby resentful. While the past action persists as a present threat against a person’s worth, it is rational to feel resentment as it challenges the implicit ‘threatening claim’ (Hieronymi 2001: 546-547). If nothing else or no one else will protest the wrongdoer’s behaviour, the victim can do so by resenting the wrongdoer.

It follows that what justifies resentment in Hieronymi’s account is not only the (justified)^11^ belief in the wrongdoer’s culpability for the offence, but also the belief that the wrong expresses or ‘poses’ a continuing threat to the victim’s sense of worth (Hieronymi 2001: 546-547). Resentment is a ‘fight response’ because it is aimed at affirming the victim’s worth (it affirms and it tries to re-establish the worth that was denied and threatened in wrongdoing) and so, when the victim forgives, it indicates that the ‘threat’ to her worth has been removed. In order to assuage the victim’s resentment, the wrongdoer should symbolically retract their ‘threat’ by apologizing. Thus, a successful apology retracts the ‘threat’ (and the insult) to the person’s worth. In apologizing, Hieronymi claims, the wrongdoer ‘renounces the deed’, and ‘anger loses its point’ (2001: 548). This explains the role of apology in forgiveness for Hieronymi: the apology retracts the insult so the victim feels safe and justified in forgiving. Similarly, Murphy maintains that by apologizing, the offender manages to ‘bring herself low’ and raise the victim up (1988: 28). In this way, the victim’s self-respect is re-established as she stops feeling threatened. The victim is now in a position to acknowledge that the offender has retracted her claim, and this recognition justifies the victim’s forgiveness because resentment is no longer warranted.

Martin (2010) summarizes the ‘insult account’ as follows: 1. Wrongful deeds insult their victim’s worth. 2. Resentment is a form of protest against this insult. 3. A successful apology retracts the insult (Martin 2010: 538). On this view, then, resentment is aimed at the insulting message expressed in wrongdoing. A good apology will negate this message and thus the victim will see that her warrant for resentment is undermined.

## Problems for the ‘Insult Account’

Martin (2010) argues that we should reject the ‘insult account’ for the following reason: this account can only be true in ‘those cases in which the wrongdoer intends to make her victim believe that he deserves his treatment or in which she tacitly believes that her victim deserves his treatment’ (2010: 541). So apology can provide a reason to forgive the wrongdoer only in a limited set of cases—those in which wrongdoing indeed sends such a message. On Martin’s view, wrongs by themselves do not have the power to insult their victims (that is, to send the message that their victims deserve no better treatment). She adopts a Gricean view of meaning and argues that what the speaker intends to communicate in uttering a certain sentence is crucial to determining the meaning of that sentence. So, on Martin’s view, one can send an insulting message only if one intends to do so. It follows that apology would make sense only in cases where the wrongdoer intends to insult her victim. However, Martin argues that apologies are appropriate even in cases where the wrongdoer has no such intention. The ‘insult account’ must therefore be wrong, according to Martin, as it cannot account for the fact that we should apologize in cases where we did not intend to insult.

However, ‘the insult account’ can reply to Martin’s criticism. Contrary to Martin’s view, wrongful deeds can have the power to send insulting messages, even in the absence of a communicative intention on the part of the speaker. For instance, I may insult someone by accident (think of a case where a person makes jokes which insult their audience, but they do not even realize there was anything problematic about that particular joke). Suppose I am chairing a session and I fail to notice when the women in the room want to speak, or maybe I fail to stop the men in the room from constantly interrupting the women when they are speaking. This incident does not merely cause inconvenience and frustration because the women are unable to express their viewpoints but, more importantly, it causes the women to feel undermined because of the institutionalized disrespect toward them. Proponents of the ‘insult account’ might reply that I need to apologize here given that I insulted the women in the room, even though I did not intend to insult them.

Nevertheless, in this paper I argue with Martin that this version of the ‘insult account’ is not satisfactory. Similarly to Martin, I agree that one of the consequences of the ‘insult account’ is that it makes sense to apologize for and to forgive only a very small group of wrongs. I agree that the wrongs accounted for are those in which the victim feels morally threatened and undermined. However, I argue that apologies are in order even when the victim does not feel undermined, so the ‘insult account’ should be modified to account for these cases as well. I will draw on the kind of distinction that Hampton makes between ‘demeaning’ and ‘diminishing’ to show how this can be done.

The ‘insult account’ proposed by Hieronymi and Murphy links the conditions of forgiveness to a claim about the psychological state of the victim’s sense of worth, specifically, feeling morally diminished (in Hampton’s sense). This, however, is too subjective a condition to ground forgiveness. Hieronymi’s account, for instance, is based on the claim that wrongdoing threatens a person’s sense of worth, and that this sense of self-worth can be redeemed in various ways. The appropriateness of forgiveness in her account therefore depends on a psychological claim about the victim’s sense of worth, rather than an objective fact about whether the victim was demeaned by how she was treated.

We can see that this is problematic in the following example. Consider, for instance, a victim of wrongdoing who is simply not the type of person to let herself be affected by moral injuries as she is confident in her self-worth. In Hampton’s words, imagine someone who is ‘beyond resentment’, in the sense that she is so confident in her standing as a person that ‘demeaning actions cannot call it into question’ (1988a: 58). Such a victim might decide to break her connection with the wrongdoer because she can see they are not pleasant, even if this is accompanied by no feeling of threat or damage to self-esteem. In this case, there is certainly a role for forgiveness. Hieronymi is mistaken to think that because the victim does not feel threatened, she does not care about the offence or she cannot be empowered to forgive. Hieronymi claims that an offence ‘fails to pose a threat only if it concerns an unimportant matter or if you fall from the status of moral peer’ (2001: 549).^12^ I disagree with her about this: if the victim morally condemns the offence and justifiably believes it is an unexcused offence, then this is sufficient for forgiveness. The victim does not need to feel a sense of threat to condemn an offence and expect an apology.

It is further unclear for the ‘insult account’ what the role of apology is in cases where the wrongdoer does not threaten the person’s sense of worth because the wrongdoer did not succeed in making the victim feel threatened. Consider a case of incomplete wrongdoing where the victim never comes to realize that she was the victim of an intended attack. For example, a murder attempt in which the would-be murderer fails to kill the person targeted (I try to shoot him, something blocks my line of sight and I fail to discharge; he never finds out about my attempt). The would-be murderer stands in need of some kind of forgiveness (apologies are also owed to the victim), despite not having managed to make a threatening claim.

If there is no insult communicated by the wrongdoing, and if the wrongdoer does not attempt to lower her victim’s value, then it is again unclear what the role of an apology is in forgiveness. On the ‘insult account’, apologies are supposed to remove the sense of threat to one’s worth and negate the insulting message that the victim deserves to be badly treated. When there is no threat or insult involved, then the ‘insult account’ cannot explain how apologies give the victim a reason to forgive. It follows that in this account it only makes sense to apologize and forgive in cases where the victim feels morally diminished. Casting the role of apology in terms of retracting a ‘threatening claim’ or an insult to one’s worth is problematic in cases where there is no such insult or threat because, as Martin puts it, ‘this means that resentment often is entirely unreasonable and that it makes no sense for the wrongdoer to ask for forgiveness or for the victim to offer it’ (2010: 541).

The fact that the victim feels hurt in these cases is not what makes forgiveness appropriate. It should instead be a normative question of whether forgiveness is in order. Where people would be entitled to feel wounded and resentful, even if they do not, forgiveness is still appropriate because they have been wronged. Likewise, an apology is not necessarily the appropriate response to a person’s sense of threat if that sense of threat is misplaced. An account of forgiveness must be able to explain why apologies give us a moral reason to forgive whenever we are wronged, and not only in cases where we feel threatened, undermined, or simply hurt.

## A New Proposal: Earned Forgiveness through Apology

I now turn to offer an account of earned forgiveness which solves the puzzle outlined in section I and which explains the reason-giving power of apology in forgiveness. I argued in the previous section that the ‘insult account’ is wrong to assume that we condemn and then forgive wrongdoers only when wrongdoers have threatened our sense of worth and then restored it, or when wrongdoing manages to insult and degrade us.

I take the view that we forgive people for wrongs done to us, and that we must adequately respond to wrongs not just because of the contingent fact that our sense of worth is damaged, *but because wrongdoing disrespects us.* I propose to understand this disrespect as objective, and follow the sense of ‘demeaning’ proposed by Hampton. I preserve here the intuition that motivates the original ‘insult account’, which is that wrongdoing insults us as persons. However, disrespectful actions are wrong even if they do not threaten our sense of moral worth. They are wrong because objectively the victims were not treated with the respect that is their due.

Apologies in my account are for wrongs done, where the nature of the wrong done is understood objectively rather than being specifically about the harm caused to the subjective feelings of an individual. I am not denying here that there is a conceptual connection between our tendency to feel hurt by certain sorts of behaviours and those behaviours counting as wrongs. Nevertheless, whether the victims are or feel diminished is not a necessary condition of apology and forgiveness. The victim of wrongdoing can recognize that her treatment has been disrespectful and blame the wrongdoer for it without feeling diminished by the moral offence.

Thus we do not need to think of resentment as necessarily responding either to a threatening claim implicit in the act (Hieronymi) or as a ‘defiant reaffirmation of one’s rank and value in the face of treatment calling them into question in one’s own mind’ (Hampton 1988a : 60).^13^ We can justifiably resent wrongdoers because they have been disrespectful toward us and thus failed to live up to certain moral norms of mutual regard. This suggests that we should separate resentment from the perception of the ‘moral threat’ to our worth and accept that we may resent wrongdoers, but not because they threaten our sense of worth: we resent wrongdoers because they do not treat us with the due respect. To illustrate the point that we should understand the wrong as ‘objective’ and not necessarily tied to subjective hurt feelings, consider an example of infidelity within a monogamous marriage. Suppose that my partner finally confesses to an affair he has had many years ago with my best friend. Our marriage was never affected by this affair and we have always had a happy marriage. Nevertheless, my natural reaction to his confession will be resentment and blame. I blame him for wronging me, thus disrespecting me during all these years, despite the fact that his wrongdoing did not affect me negatively in any way.

This idea that we resent wrongdoers for the disrespect and lack of consideration expressed in their actions reflects the view of wrongdoing proposed by Gaita (2004), Darwall (2006), Strawson (2008) and Smith (1982). Gaita (2004),^14^ for example, argues that what really matters to us when we are wronged is not only the natural harm (physical injury, inconvenience, the loss of possessions, etc.) caused by the wrong done to us, but that we have been wronged as persons. This relates to the Kantian idea that it is important for us to be treated and recognized as moral equals in the moral community as we all have equal worth, so we care about relations of moral equality between people. If a black person is not allowed to enter a shop because she is black, she is not *only* concerned by the disutility of being unable to buy from that shop, but more importantly because she was insulted as a person: she is *demeaned* by the fact that she was not treated with the due respect or with the correct value as a person. Darwall (2006) argues this by suggesting that moral wrongs are mainly wrongs to our person:

When someone uses your foot as his footrest, this is an injury not just to your foot, but also to your person. It is a failure to respect your standing or dignity as someone who may not be so treated and who has the standing as one among others to hold others to this. (2006: 84)

Thus, whenever there is wrongdoing, whether my partner has betrayed me or someone has hacked my email account, all these wrongs do in fact involve a failure of respect of some kind. Of course, wrongs involve many other things like hurt, disappointment, loss of trust and so on, but they also necessarily involve a failure of respect. Following this idea, I propose to understand wrongdoing as expressing disrespect for the victim and therefore creating *a deficit of respect.* Thus there is a necessary task to be done after wrongdoing: the disrespect shown must be corrected and the victim’s respect must be reaffirmed. The wrongdoer must recognize that how she treated the victim was wrong, that she should not have treated her in such a manner, and that the victim deserves to be treated better, with more respect. I must add at this point that I do not assume here that apologies will repair all the damage involved in wrongdoing. My claim is modest: the apology will redress the mistreatment involved in wrongdoing; the wrongdoer now treats the victim as someone who is owed respect, as someone who is unacceptable to wrong again. However, often apologies will not be sufficient to address most of the other elements involved in an act of wrongdoing. So the wrongdoer might have to compensate the victim or her family, incur punishment, work for the community, properly engage with the victim’s hurt feelings and make up for any physical or material harm that wrong has caused.

Let me explain my model by using an example. Suppose your friend Bill gossips about you at a party and spreads malicious rumours behind your back. In this case, Bill’s action expresses disrespect, so that the balance of respect between you and your friend has you in deficit. The question is what can be done to make good the deficit of respect that has been created by Bill’s betrayal? The answer is a meaningful apology on the part of your friend. This suggests that when someone accepts a meaningful apology, it is because the deficit of disrespect has been made good by the apology, and thus forgiveness can be forthcoming.

Forgiveness indeed involves some revision in judgment, as Hieronymi argues. However, unlike in Hieronymi’s account, what is happening when we forgive is that we are giving up the judgment that the wrongdoer continues to disrespect us. The fourth condition for appropriate forgiveness thus should not be that the victim’s sense of worth has been threatened and rebuilt, but the fact that the wrongdoer has changed the balance of respect: there is no longer a deficit of disrespect. It is this objective, expressed disrespect that is made good in apology, not any subjective feeling of being morally diminished.

Following an apology, personal relations will normally be restored as well. However, it is not possible to have this intimate restoration of personal relations without first restoring the more generic moral relationship.^15^ Earned forgiveness restores the moral relationship between the victim and the wrongdoer following an apology because the apology restores the expressed levels of respect to one of equal respect. Apology does this because the wrongdoer distances herself from the wrong action by morally condemning it and, by dissociating herself from wrongdoing she avoids complicity and acquiescence.

Of course, in certain cases of wrongdoing it may be decided that, given the situation, it is better if people continue to be estranged from one another. In these cases there is a sense in which the relationship is not totally restored, as the individuals feel they cannot be intimate again as friends. Nevertheless, apology does its job as moral respect is equalized even in this case: it is possible in forgiveness to restore the moral relationship and decide not to restore the personal relationship.

So far, I have argued that in forgiving the victim acknowledges that the offender has made good the deficit of respect. I will now argue that forgiveness further involves a commitment to repair relations with the wrongdoer. This commitment is justified by the fact that the wrongdoer, by apologizing, has made good the existing deficit of respect, and in so doing has restored the moral equality that was lost in wrongdoing. In a minimal sense, what forgiveness restores is the moral equality between the victim and the wrongdoer that I understand in terms of the ‘reciprocal recognition of the standing to make certain demands of one another, that is, in the moral case, mutual respect of the equal dignity of free and rational persons’ ( 2006: 83-84). Thus, earned forgiveness acknowledges that respect relations between the victim and the wrongdoer have been restored and this, in turn, is followed by a commitment to repair relations with the wrongdoer.

My account solves the puzzle of forgiveness because the victim continues to believe that the wrongdoer is culpable for the wrong done, but the balance of respect between the two has nonetheless been restored. Earned forgiveness is justified by moral reasons: forgiveness is appropriate following an apology because the respect score was changed so victim and wrongdoer are on a par again.

One of the reasons my account is superior to the original ‘insult account’ is that it opens up the possibility that, although an apology has to express remorse and forgiveness typically overcomes, to a certain extent, negative feelings (an apology justifies this, but it will always remain open to the victim whether she relinquishes blame feelings), nevertheless an apology need not express thoroughgoing remorse and forgiveness need not overcome all resentment.^16^ The original ‘insult account’ is committed to the implausible view that you can only apologize when you are remorseful in a thoroughgoing way, because only then can you undo the threat. It is also committed to the view that negative feelings of resentment need be totally overcome in forgiveness.

I can count as forgiving you and I can sincerely communicate my forgiveness to you even if my heart has not quite caught up with the emotional perspective I have committed to. This is because at an expressed level there has been an equalization of moral relations between us. That is to say, the deficit of respect that you brought about by wronging me, you have now made good again by apologizing, and therefore I can acknowledge this in forgiving you.

The moral relationship is restored in apology and in forgiveness primarily at the expressive level through a declaration that each party is equally morally worthy. My account therefore improves on Hieronymi’s account because an apology can do the moral work and can justify the victim’s forgiveness even if her emotions have not yet properly caught up.

In my model, the necessity of apology for earned forgiveness is based in the wrongdoer’s recognition of the necessity to make good the deficit of respect caused by their wrongdoing. This can be done symbolically by admitting that the victim was not treated with the due respect, however, in many cases, the offender should also compensate the victim for the inconvenience caused or any material harm brought about by the wrongdoing. Further, it is important to make good the experienced deficit of respect, both when the victim is not emotionally affected by the wrongs against her, and even when the victim continues to feel diminished for an unduly long time. We can do justice to the fact that forgiveness is grounded in moral reasons if we argue that what gives us good moral grounds to forgive is not the alleviation of a sense of threat or the fact that the wrongdoer has undergone humiliation in begging for forgiveness (Murphy), but making good an objectively existing deficit of respect caused by the wrong done.

## The Role of Apology in Earning Forgiveness

My view of wrongdoing and forgiveness fits very well with an existing account of apology proposed by Bovens (2008).^17^ The logic of wrongdoing and forgiveness implies that we need an account which portrays apology as a central kind of speech act which is capable of redeeming the deficit of respect involved in wrongdoing. I have argued that apology is an appropriate response to the acceptance that one is a wrongdoer, for the purposes of correcting for the disrespect expressed. I have also claimed that apologies do more than merely signal the extinguishing of a threat. Apologies have reason-giving powers: they justify forgiveness.^18^ What, then, is the role of apology in forgiveness and why does an apology give the victim a reason to forgive?

I suggest that we can make sense of the claim that apology can equalize the level of respect by understanding apology as a speech act whose illocutionary point is that of affirming respect for the victim. Apologies, Bovens claims, are ‘admission that I did not treat you with the respect that is due to you. I bow my head to make up for the deficit of respect in my earlier treatment of you’ (2008: 231). Bovens uses an example from Kant to illustrate this idea. Imagine a case where a rich offender, in apologizing, kisses the hand of the victim who is lower in social status. Through this gesture of humility, the wrongdoer expresses an ‘excess of respect, and this excess is meant to put the scales of respect back into balance’ (Bovens 2008: 231). Thus, for Bovens, it is a necessary condition for a successful apology to show excess respect to the victim.

Although I am sympathetic to Bovens’ view, I am not committed to the view that apologies work because the wrongdoer expresses ‘excess respect’ for the victim. Contrary to what Bovens suggests, we cannot literally make up the deficit by quantitatively adding a bit more respect so that the victim is not in deficit anymore. One can apologize, and this apology would redress the balance of respect, but without paying ‘excess respect’ or by showing an excess of humility. Let us now see why an apology has force. Apologies typically involve four elements^19^:

1. The acceptance that one has done wrong.^20^
2. An admission that the wrongdoer did not treat the victim with the due respect.^21^ I add to Bovens’ analysis that this will be followed by an attitude of self-blame: the wrongdoer now stands by the victim, admitting that the victim was disrespected and thus reaffirming the victim’s moral standing. Self-blame involves repudiating the wrong done by committing the wrongdoer not to repeat the moral offence. For moral wrongs that result from characteristic bad behaviour,^22^ self-blame means that one is committed to change the character traits that made possible the moral offence. Further, the disposition not to repeat the moral offence is part of the sincerity conditions of a successful apology, and this partly explains why the balance of respect is restored following a sincere apology.
3. Apologies imply a certain sort of moral risk. The wrongdoer takes a risk by giving the victim the opportunity and the power to decide whether the wrongdoer can be accepted back as a person worthy to associate with on equal footing. Bovens claims that by transferring this power to the victim, the wrongdoer expressively pays excess respect to the victim. By admitting her fault, the wrongdoer ‘acknowledges a loss of moral stature due to her wrongdoing. She can regain this moral stature only if the victim freely awards her the respect that is due to her on grounds of her personhood’ (Bovens 2008: 233).
4. They commit the offender to repair the harm (the inconvenience caused, etc.) occasioned by the wrongdoing in a proportional manner.

I would like to develop Bovens’ idea that apology redresses the balance of respect, and claim that apology does this in a unique way. That is to say, I argue that the wrongdoer cannot make good the deficit of respect without apologizing to the victim, and it is this unique role that an apology has which can earn the wrongdoer’s forgiveness. At first, this claim may seem counterintuitive. Suppose A has insulted his friend B by unjustifiably doubting B’s philosophical abilities and by not inviting B to speak at a conference A organizes, despite his initial promise that B will be one of the invited speakers. Now suppose that later on, A changes his mind and decides, nevertheless, to invite B to the conference as a plenary speaker, but with no intention to apologize. Does not this make good the deficit of respect, even if A has not apologized? The answer depends on how we interpret A’s attitudes toward B, that is, on whether A’s intention was to apologize.

If we interpret A’s gesture as an apology, then it does manage to redress the balance of respect, precisely because it is an apology. The forms of expressing and communicating an apology are not fixed—what is important is that an apology be expressed in some way or another, no matter whether it is done through deeds or words. If A’s gesture of inviting B to the conference does not count as an apology (suppose A only wants to make B feel better and boost his confidence, but has no intention whatsoever to admit he was wrong), then it cannot redress the balance of respect. The wrongdoer has to show respect to the victim *with regard to that particular wrongdoing*, by admitting that he was wrong about that offence. A can only make good a particular deficit of respect if A invites B to the conference in order to make up for the fact that A has wronged B with that particular offence in mind. If it simply happens that A invites B to the conference because there are not enough speakers, then it is not an apology and it has not made up the deficit of respect because it is not making up the deficit of respect caused by that insult. A has to make up for the deficit of respect to B in relation to that offence which caused the deficit in the first place.

So it seems that apology is effective in forgiveness because it can be related to the victim in the right way: the wrongdoer shows the victim due respect by admitting wrongdoing. Apology is an act where the offender relates to the victim in a respectful way, as she should have done in the first place. The offender’s focus in apology is on the victim of wrongdoing and on that particular offence, and not on oneself or on other factors irrelevant to the wrong done. Consider for example a self-indulgent way of showing regret about a particular wrong.^23^ Imagine a case where you encounter an old friend of yours who once bullied you for being overweight. She has now changed to the extent that she cannot believe that she could have behaved so abhorrently. She is now ashamed because she thinks this is not how a decent person should treat people. She regrets her old character but she fails to consider how much she has offended you in particular (she is not focused on the deficit of respect caused by her insult) and so she never apologizes.

Similarly, imagine a case where the same friend is repentant before God. She cares about not having followed her obligations toward God at the expense of a proper acknowledgment of you, as her victim. She might have a grasp of the seriousness of the situation but, again, fails to appreciate how much she has hurt the victim. Although your friend has provided you with evidence that she has changed, you might reasonably feel offended by her failure to apologize and you might consider it inappropriate to forgive her.

The wrongdoer has failed to earn forgiveness in these cases precisely because the offender is not properly focused on the deficit of respect caused by that particular offence. The deficit of respect which needs to be made up for is a specific deficit of respect caused by that wrongdoing. It is not about net levels of respect which could be counterbalanced in different ways. It is not just a matter of showing the victim you respect her generally, but of addressing and engaging with the particular insult which was expressed in the wrong done. When A invites B to the conference, it shows that A has enough respect for B to do that. Maybe A has changed his mind about B and he thinks B is a good person to invite. However, it still does not redress the balance of respect as it does not address the initial insult. A’s invitation only shows that A respects B, but without actually correcting for the deficit of respect caused by the fact that A has unjustifiably doubted B’s competence to begin with, as A does not apologize for that act.

We are now in a position to see clearly that the ‘insult account’ as others have formulated it, by claiming that after wrongdoing the victim’s self-esteem must be restored, does not do enough to explain that restoring the victim’s self-esteem must be in respect of the particular wrong which needs to be made good. In the previous section, one line of objection was that, on the one hand, the ‘insult account’ is too dependent on the psychology of the victim and thus on a subjective understanding of “wrongdoing”. On the other hand, we can see that it does not make it clear *enough* that it is that particular wrong that needs to be made good, and not just net levels of respect. My complaint then is not only that the ‘insult account’ is not normative enough, but also that it is not focused enough.

An apology needs to be communicated to the victim in some form in order to earn forgiveness (my justified belief that my friend is sorry for betraying me is not enough to earn my forgiveness). This is because the force of an expressed apology is that it is performative, and thus it expressively corrects for the balance of disrespect. When we apologize we change the moral relation with the victim and our commitments toward her (the wrongdoer commits to repair relations with the victim) by affirming the fact that she is owed better treatment from us.

This suggests that apology is the appropriate frame for communicating our remorse as it encourages proper, non-corrupt forms of remorse. As I have argued, it is properly focused on the victim and on the wrong done to the victim, and thus it can achieve its point of expressing the moral respect owed to the victim, in relation to the specific deficit caused by the wrongdoing. So my aim here has been to propose an improved version of the ‘insult account’ that offers guidance on how a theory of earned forgiveness can successfully solve the puzzle of forgiveness while explaining the important (but not necessarily exclusive) role that apology plays in justifying and earning forgiveness. There are, of course other ways that forgiveness might be justified, but not earned, by the wrongdoer. So apology is not the only justification for forgiveness, but it is the only way that the wrongdoer has the power to give the victim a reason to forgive.

I have proposed to understand earned forgiveness as a commitment to repair relations with the wrongdoer, grounded in a change of judgment, where that change of judgment is a response to the fact that the wrongdoer has corrected the balance of respect by making up for the disrespect expressed by the wrong done. The normative account I have defended does justice to the fact that forgiveness is sensitive to moral reasons; the wrongdoer apologizing to the victim justifies it. Finally, my account can explain the reason-giving power of apologies in forgiveness: they make good the particular deficit of respect expressed by the wrong done.

## Concluding Remarks

By way of concluding I would like to consider a possible objection to my account. Hallich (2016) has recently argued that there is an inherent paradox involved in every case of apology, which undermines the moral status of apologies. For Hallich, when a wrongdoer apologizes, she also necessarily asks to be forgiven. This in turn means that the wrongdoer, when she apologizes, implicitly seeks to alter the victim’s justified blame feelings; she hopes the victim will relinquish negative feelings towards her. Hallich claims that a perpetrator ‘offers his apologies to bring about such an alteration in the victim’s feelings. If this were not the case, there would be no reason for the offender to address the victim at all’ (2016: 1009).

Furthermore, Hallich argues, there is a certain tension in the perpetrator’s request for forgiveness. If his apology is sincere, he surely should accept that the victim’s negative feelings are justified. Moreover, feeling remorse about one’s action further implies that the wrongdoer himself condemns his actions, so he shares the feelings and the moral understanding of the victim. The wrongdoer is himself indignant about what he has done and he himself wants to repudiate his wrongdoing. But if he accepts that the negative feelings against him are *still* justified, then he should not ask the victim to relinquish them. This is how Hallich describes the paradox of apologies:

Why, then, should he want to alter the victim’s feelings of resentment? By apologising, the offender tries to bring about a state of affairs which, if genuinely repentant and remorseful, he has no reason to want to bring about. (2016: 1009)

My reply to Hallich’s concern is as follows. It is not true that a wrongdoer’s need to apologize comes from the fact that she needs to alter the victim’s feelings of resentment, even if this may be a desirable consequence. A wrongdoer may hope to achieve forgiveness, but without necessarily demanding it. It will remain open to the victim whether she accepts the apology. The need for an apology comes first from the fact that one needs to disapprove of wrongdoing and condemn wrongdoing. Otherwise, the wrongdoer’s silence might be interpreted as moral condonation or even complicity in wrongdoing. By apologizing, the wrongdoer performs the symbolic act of disavowing and distancing herself from wrongdoing. In order to agree with the victim and share her condemnatory feelings or attitudes towards the wrong done, one must first admit she has done wrong.

Of course, one can admit wrongdoing without communicating this to the victim. As Hallich acknowledges, you can be truly sorry and change your attitudes towards someone, without necessarily communicating this to the victim But I disagree with Hallich here. Apologies must be communicated in order to achieve their expressive and symbolic point, although not necessarily by uttering the words ‘I apologize’. It is enough if the wrongdoer performs some symbolic gesture with the intention to apologize and which, in turn, is meaningful to the victim. Moreover, a wrongdoer has to express her apologies even in cases where there is no hope for forgiveness—for example, due to the fact that the wrong done is unforgivable. There is a further reason to apologize: the wrongdoer has to show her moral solidarity with the victim of wrongdoing, as she owes something to the victim. I agree with many writers that ‘violations of rights must be given proper recognition’,^24^ and a failure to publically blame wrongdoer (or publicly apologize, when one is a wrongdoer) shows our indifference or the perpetrator’s indifference towards the gravity of the wrong done.

In the view I put forward, apologizing is not simply a matter of whether the wrongdoer is rationally entitled to do it, but it is a matter of what he is required to do as a matter of fairness. It is not the case that, at least sometimes, we should ‘see the refusal to ask for forgiveness as a virtue rather than as a vice’ (Hallich: 1020). The offender has to correct for the particular deficit of respect he brought about in wronging the victim, and thus he has to retract the insult involved in wrongdoing, despite the fact that he now shares the point of view of the victim. The deficit of respect cannot be made good by remorse which is not expressed in any way, but merely privately experienced. Apology has an expressive power and it has to be communicated in order to affirm the necessary respect for the victim of wrongdoing and thus to counterbalance the disrespect expressed in wrongdoing. Surely, apology may not be enough to totally repair the wrong done; the wrongdoer may need to make further amends, depending on the nature of the wrong and on the relationship he has with the victim. However, apology is a necessary step for at least starting to redress the wrong and making it safe for the victim to forgive. In the absence of an apology, the victim might feel she was not even offered the chance to engage with the wrongdoer, and thus maybe her only opportunity to move on with her life has been taken from her.

Another difference between my account and Hallich’s is that I am not claiming that, in accepting an apology, the victim needs to entirely relinquish all her resentful feelings right away. So accepting an apology is compatible with continuing to resent the wrongdoer. I follow Butler’s (2006) suggestion on this and agree with him that, even after the victim has forgiven, some form of justified resentment may remain in us.^25^ As I claimed earlier in the paper, forgiveness is indeed incompatible with seeking revenge for the wrong done, or feeling disproportionate resentment and anger which will impede a normal relationship between the victim and the wrongdoer based on good-will and respect; however, some level of negative feelings may still be justified and reasonable to have, and having these feelings is compatible with experiencing genuine forgiveness.^26^ Thus, when the victim accepts the wrongdoer’s apologies, what she accepts is the fact that the wrongdoer has retracted her disrespectful claim and she is now committed to treat the victim with the respect she is owed. However, this does not mean that the victim will automatically overcome her justified resentment, and she is not expected to do so. When a wrongdoer apologizes, she is not necessarily trying to convince the victim to modify her justified moral emotions.

One implication for my account is that the metaphor ‘wiping the slate clean’, typically associated with forgiveness, doesn’t rightly characterize what we do when we forgive. Forgiveness cannot imply that we continue the relationship with the wrongdoer in the same way as it was before the wrongdoing. My view of the wrongdoer and the way we restore our relationship will still be affected by the way she treated me. However, when we forgive we do commit to put the wrong behind us in the sense of not bringing it up again in future conversations in a condemnatory manner, for example. Forgiveness is surely incompatible with reminding you, every time you do something wrong, that you also wronged me last year. If I forgive you for what you did to me last year, then it would be cruel of me and irrational to keep bringing up that argument again in our conversation. So in forgiving we relinquish our right to harbour blaming attitudes toward the wrongdoer, at least concerning that particular offence.

## Footnotes

1 For a detailed defense of the view that forgiveness involves a certain commitment towards the wrongdoer (not to return to the previous state of resentment), see Scarre (2016).

2 See Pettigrove (2012) and Hallich (2013).

3 Bennett (2003) argues that there are two types of forgiveness, ‘personal forgiveness’ which is a form of elective forgiveness offered without apology, and ‘redemptive forgiveness’, which is justified by the wrongdoer’s apology or remorse.

4 Pettigrove (2012) argues that there is a kind of forgiveness which need not be earned, what he calls ‘gracious forgiveness’.

5 For a similar view, see Haber (1991), Norlock (2009) and Pettigrove (2012).

6 Recently philosophers have argued that there is in fact a broad class of emotions that forgiveness overcomes. See for instance Pettigrove (2012) and Walker (2006).

7 Some authors argue that if the victim tries to excuse the wrongdoer’s behaviour then this may fall into complicity and condonation of her actions, because it would absolve the wrongdoer of culpability, and therefore it does not count as forgiveness. For this idea see Kolnai (1973), H.J.N. Horsburgh (1974), Hieronymi (2001), Bennett (2003), Murphy (2003) and Zaragoza (2012).

8 See Murphy (1988), Hampton (1988), Haber (1991) Hieronymi (2001), Griswold (2007) and Bell (2012).

9 See for example Bell’s (2012) account of the conditions an apology needs to meet in order to provide an overwhelming reason to forgive.

10 I talk here about earning forgiveness but I dispute the assumption that earning forgiveness through apology will automatically generate obligatory reasons to forgive. One can earn forgiveness and this ‘earning’ may only generate permissive reasons, rather than obligatory reasons. This doesn’t mean that in the absence of an apology, the victim’s forgiveness will be impermissible. I am pluralistic about the sorts of reasons that justify forgiveness: apology can justify forgiveness but so too can non-merit-based reasons. So elective forgiveness will be justified by reasons which do not come from apology or remorse, but which are related to the moral character of the victim (see Pettigrove 2012 and Hallich 2013 for other non-merit based reasons which can justify elective forgiveness). One may also argue that none of these reasons have the power to generate moral obligations. It will remain open to the victim whether she will forgive her wrongdoer or not, even if these reasons will count in favour of forgiving the wrongdoer.

11 There is an interesting question here of whether the belief in the culpability of the offender must be justified or not. I think the belief must be justified to constitute justified resentment. What justifies my resentment is the fact that the wrongdoer is culpable. So, resentment should be justified by a justified belief in the wrongdoer’s culpability. Nevertheless, it is not clear if Hieronymi leaves open the possibility that even an unjustified belief could justify one’s resentment. Sometimes she seems to use more internalist terms when she talks about the judgments that justify resentment.

12 See also her reply to Hampton and Minas in footnote 34 where she seems to suggest that if we care about human beings we will feel threated about actions that disrespect us.

13 On Hampton’s view, resentment is never a justified feeling to have, following a wrong. This is because she understands resentment as a fight response, a *personal defence*, which hides insecurity and which tries to reaffirm the victim’s superior status relative to the wrongdoer, so it is never justified. For a different view on resentment and its justification, see for instance MacLachlan (2010).

14 See the ‘Preface to the second edition’.

15 In the case of strangers, the only possible relationship to be restored would be the moral relationship. Scanlon (2008) uses the idea of the ‘moral relationship’ to explain blame of strangers.

16 My account is compatible with the view that forgiveness is possible in the absence of negative feelings (the victim blames the wrongdoer for what she has done, but she does not experience any negative feelings). Further, it is compatible with the victim’s partial overcoming of resentment, which can, nevertheless, return when the victim reflects on what happened to her. So I can express a moral commitment towards the wrongdoer by uttering “I forgive you”, for example, even if I still harbor negative feelings towards her. Other philosophers who argue that forgiveness can consist in a performative utterance are Haber (1991), Norlock (2009) and Pettigrove (2012). For example, Haber compares the performance of ‘I forgive you’ with the utterance of a promise. Thus, according to him, ‘I forgive’ you can be a successful speech act even if it turns out the victim finds herself harboring anger when she thinks again about the wrong done to her at later times. It is important to distinguish my account from accounts that argue that forgiveness is a process rather than an act. Horsburgh (1974) argues that forgiveness is a process and thus we can say we have forgiven another even if we have not totally overcome our negative feelings toward the wrongdoer. However, he thinks that the forgiveness process is not complete until all feelings of resentment are overcome. My account is different from this as I agree with Butler here that genuine forgiveness is compatible with some form of negative feelings towards the offender.

17 I could equally have used the well-known account of Smith (2005, 2008). However, there are elements in Bovens’ account that are particularly apt for my discussion and which fit perfectly the balance of respect account I propose here.

18 On this point, see Bell (2012).

19 Smith (2005) proposes nine elements that an apology must have in order to count as ‘categorical’; that is, a full apology. See also Smith (2008).

20 According to Bovens, ‘an admission of a moral failing’ (Bovens 2008: 231).

21 On this point see also Pettigrove (2012: 119) and Radzik (2009: 92–97).

22 See Pettigrove and Collins (2011).

23 Gaita (2004) also discusses corrupt forms of remorse, especially egocentric corruptions.

24 See Scanlon (2003) and Bennett (2013) on this point. Bennett, for example, argues that ‘a failure to dissociate from the wrong represents culpable indifference’. (p. 80)

25 Butler portrays forgiveness as a correction of our negative and exaggerated feelings of resentment towards the wrongdoer. He says that when we are wronged, we may fall into an ‘extravagant and monstrous kind of resentment’ because we are not capable of impartially thinking about the wrong done to us and we make too much of it (p. 93). While resentment is a virtue and has been implanted in us for a good reason, it can become a vice when it is transformed into malice and revenge. This is where forgiveness finds its place. Forgiveness corrects for the ‘excess and abuse’ of the natural feeling of resentment (p. 99). For a similar view of forgiveness, see Hampton (1988a, b) and Bennett (2003). These accounts argue that we tend to exacerbate our negative feelings towards the other person; we misperceive what has happened and we tend to see the other person as totally reduced to this particular wrong. These feelings, in turn, are disproportionate to the wrong done to us, which is why they are unjustified. The role of forgiveness then, in these accounts, is to do justice and correct for these exaggerated feelings, so we can again see the wrongdoer in a more positive light, with a corresponding attitude of good will.

26 Hallich distinguishes between genuine forgiveness, where resentment is totally overcome, and ‘quasi-forgiveness,’ where we still resent the wrongdoer, but we abstain from expressing our resentment towards them.
